# Impending Paradoxical Embolism Traversing Three Cardiac Chambers Presenting With Stroke and Pulmonary Embolism

**DOI:** 10.7759/cureus.9416

**Published:** 2020-07-27

**Authors:** Nihar Jena, Ramzi Ibrahim, Deepthi Tirunagari, Kirit Patel

**Affiliations:** 1 Internal Medicine, Saint Joseph Mercy Oakland Hospital, Pontiac, USA; 2 Cardiology, Saint Joseph Mercy Oakland Hospital, Pontiac, USA

**Keywords:** impending paradoxical embolism, pulmonary embolism, stroke, thrombus, patent foramen ovale

## Abstract

A straddling thrombus within a patent foramen ovale (PFO), also known as an impending paradoxical embolism, is an infrequent event that is rarely encountered in clinical practice. This should be considered whenever there is an arterial embolism in the presence of right-sided thromboembolic disease. Symptoms are widely variable depending on the severity of thrombus and obstructive embolic events. We present a patient who arrived at the hospital with signs and symptoms consistent with a cerebrovascular disease that was ultimately diagnosed with small foci of acute ischemic cerebral infarcts, bilateral pulmonary emboli, and a straddling thrombus traversing three cardiac chambers. Treatment included PFO closure, inferior vena cava filter placement, and surgical thrombectomy with a successful outcome. Consensus for treatment of an impending paradoxical embolism in the medical literature is a subject of controversy and is our reason behind contributing this case presentation with our treatment strategy.

## Introduction

Paradoxical embolism, an underdiagnosed etiology, by definition, is an embolism that originates within the venous circulatory system that enters the arterial system. An arterial embolism secondary to a paradoxical phenomenon occurs less than 2% of the time when compared to other causes of arterial emboli. The two most common causes for a paradoxical embolism include patent foramen ovale (PFO) and an existent arteriovenous malformation. PFO is a common functional intracardiac shunt attributing to 25% of the adult population [1]. The majority of individuals who have a PFO are asymptomatic and do not require closure. A straddling thrombus in a PFO is an infrequent event that is termed as an impending paradoxical embolus (IPDE) and necessitates immediate recognition [2].

In this case, we present a patient who acquired paradoxical emboli from a deep vein thrombosis (DVT), resulting in pulmonary and cerebral emboli, and an intracardiac thrombus that transverses three different heart chambers. The intracardiac thrombus began in the right atrial cavity and crossed into the left atrium via a PFO and ended in the left ventricle through the mitral valve. This IPDE was distinctively immense and recognizable through a transesophageal echocardiogram (TEE), as shown in this case report. 

## Case presentation

**Figure 1:**
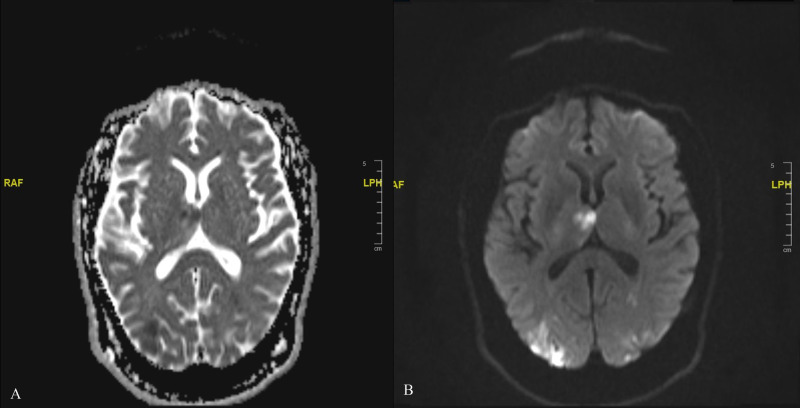
MRI of the brain of showing multiple embolic stroke (black arrow). (A) Hypointensity signals in an apparent diffusion coefficient (ADC) image. (B) Hyperintensity signals in a diffusion-weighted image (DWI).

A 65-year-old male was admitted to the hospital for a two-day history of confusion, slurred speech, and right upper and lower extremity weakness. Past medical history is significant for hypertension. His vital signs showed an elevated blood pressure of 166/111 mm Hg. Physical examination was remarkable for mild right upper limb motor weakness with pronator drift and right facial droop sparing forehead. His initial National Institutes of Health Stroke Scale (NIHSS) was 3. His lab work was significant for an elevated troponin I level of 17.27 ng/ml, B-type natriuretic peptide was 191 pg/ml, and prothrombin time was 13.6 seconds, while other laboratory findings were within normal limits. CT scan and, subsequently, MRI of the brain were obtained, revealing multiple small foci of acute ischemic infarcts with lesions of hemorrhagic transformation in various locations, consistent with microemboli (Figure 1).

**Figure 2:**
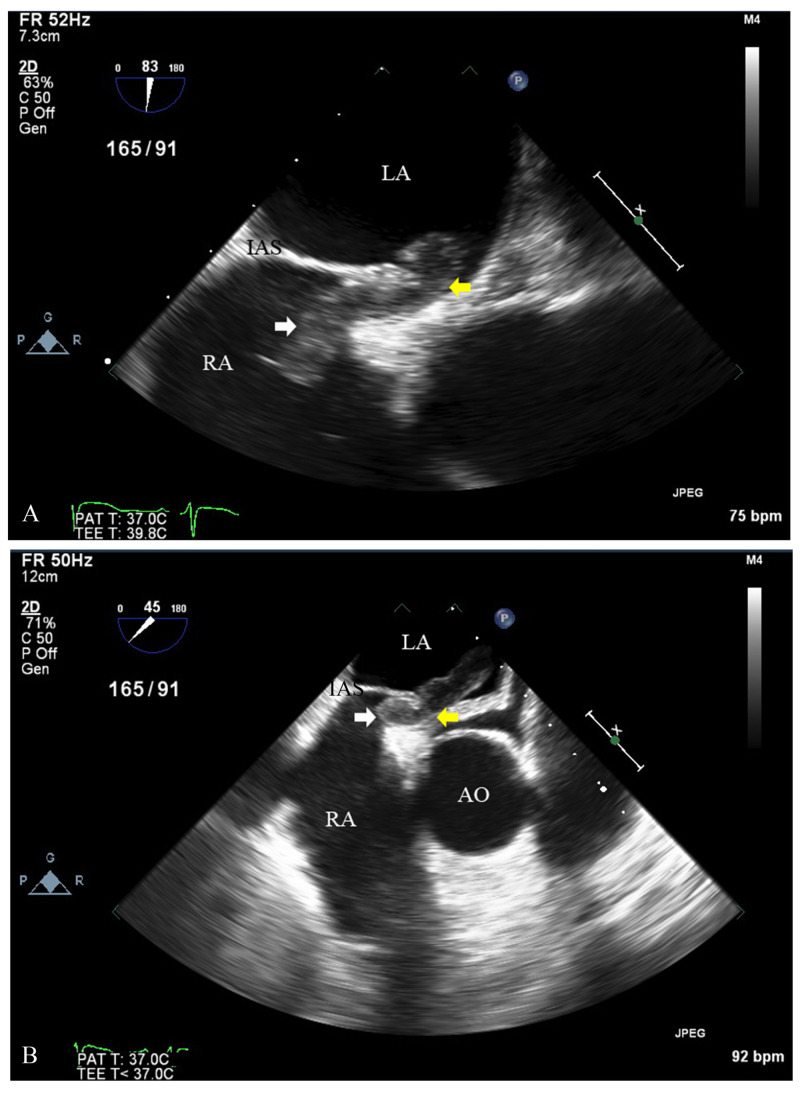
(A, B) Transesophageal echocardiogram showing a short-axis view of the aorta with thrombus (white arrow) migrating from right atrium to left atrium through patent foramen ovale (yellow arrow). RA, right atrium; LA, left atrium; AO, aorta; IAS, interarterial septum.

**Figure 3:**
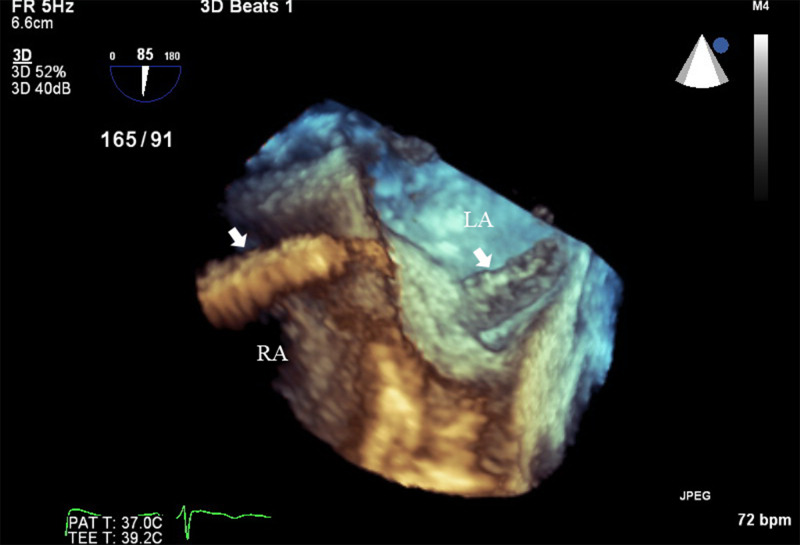
Transesophageal echocardiogram showing a three-dimensional image with thrombus (white arrow) migrating from right atrium to left atrium through patent foramen ovale. RA, right atrium; LA, left atrium.

Considering multiple shower emboli and watershed involvement, a cardiac source of thromboembolism was high on the differential. A transthoracic echocardiogram (TTE) followed by a TEE was performed, showing a sizeable mobile thrombus that appeared to begin in the right atrium extending through the PFO into the left atrium, exiting into the left ventricle through the mitral valve (Figures 2, 3; Videos 1-3). 

**Video 1:** Transesophageal echocardiogram showing a short-axis view of the aorta with thrombus migrating from right atrium to left atrium through patent foramen ovale.

**Video 2:** Transesophageal echocardiogram showing a mid-axial view with thrombus migrating from the left atrium to the left ventricle through the mitral valve.

**Video 3:** Transesophageal echocardiogram showing a three-dimensional view with thrombus migrating from right atrium to left atrium through patent foramen ovale.

**Figure 4:**
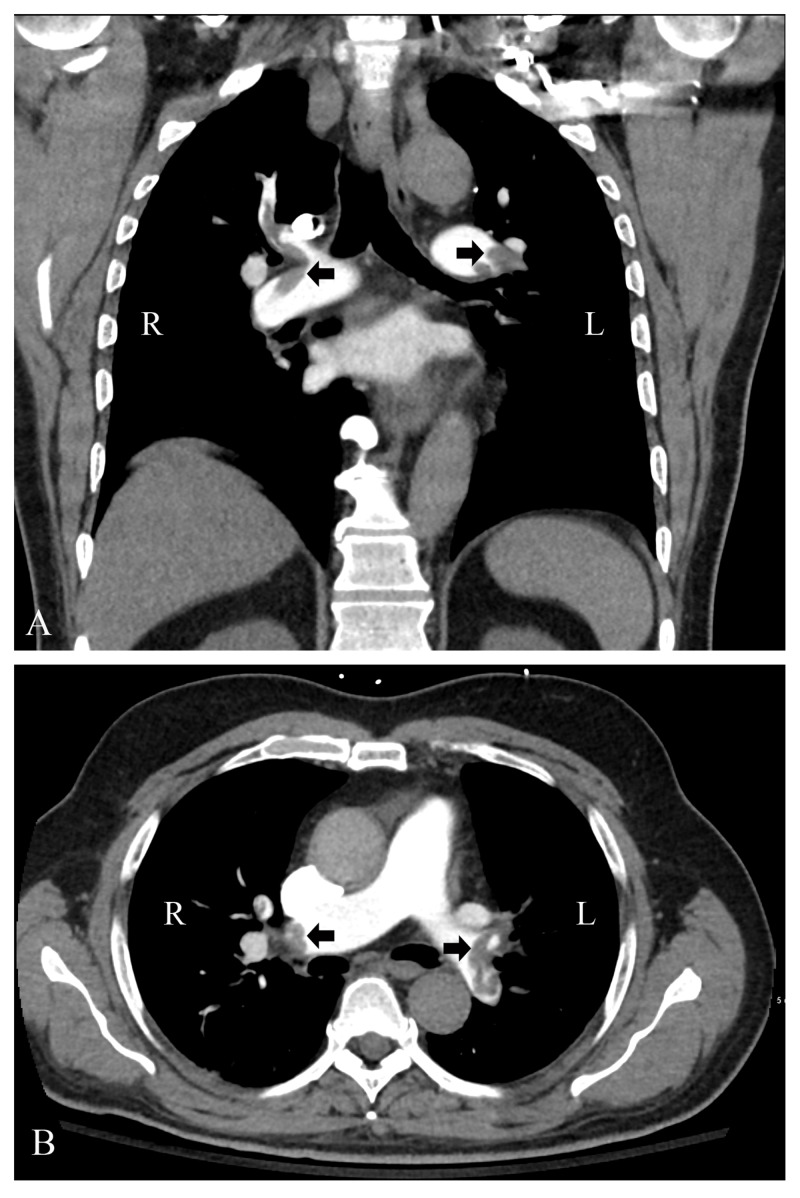
CT angiogram of chest. (A) Coronal view and (B) axial view showing bilateral filling defects representing pulmonary embolism. R, right lung; L, left lung.

Further assessment with CT angiography of the chest showed pulmonary emboli (PE) within bilateral pulmonary arteries (Figure 4).

DVT located in the left common femoral and profunda veins was appreciated on the Doppler ultrasound. A diagnosis of cardioembolic stroke due to IPDE and PE was made. Tissue plasminogen activator was deferred in our patient due to hemorrhagic transformation. He was started on intravenous heparin. Surgical evacuation of cardiac thrombus with the closure of PFO and left atrial appendage ligation was completed under cardiopulmonary bypass. An inferior vena cava (IVC) filter was placed. The retrieved thrombus was 13 x 0.6 cm in dimension and sent in for histopathology.

## Discussion

A paradoxical embolism is a presumptive diagnosis that occurs upon passage of thromboembolism that courses into the arterial circulatory system from a venous pathway, usually through a PFO or arteriovenous fistula. This is an infrequent occurrence and a less common cause of arterial embolism. IPDE is a rare condition where a thrombus straddles the foramen ovale with a high risk of arterial embolism and is associated with a higher mortality rate when associated with a PE [3]. This finding is seldom visualized, but in our case, it was demonstrated by TEE as it began in the right atrium, crossing into the left atrium and into the left ventricle through the mitral valve.

Careful assessment and urgent treatment were done immediately to avoid detrimental complications. In our patient, the management included a surgical thrombectomy and immediate anticoagulation therapy due to already existent PE. Surgical intervention can be used in conjunction or substituted with thrombolysis or anticoagulation therapy. A literature review by Myers et al. described surgical thrombectomy as having a superior outcome to other methodologies, attributed to reduced systemic thromboembolism and improved survival in comparison to anticoagulation alone. On the other hand, thrombolysis showed an opposite trend [2]. Additionally, a case report was written in 2006 by Can et al. showed improvement in patient status without embolization and complete disappearance of IPDE after 10-day therapy with anticoagulation [4]. However, this patient did not undergo surgical thrombectomy due to metastatic breast cancer along and stable hemodynamic status, unlike our patient, who already experienced systemic embolization. Finally, an IVC filter was placed in our patient due to the high risk of further embolization events. This was supported by Meacham et al., who recommended an emergent intracardiac embolectomy, PFO repair, and an IVC filter placement [5]. Also, this was supported by the PREPIC1 trial, which showed the effectiveness of IVC filters in patients being treated with concomitant anticoagulation, showing a successful reduction of PE [6,7]. Historical guidelines for an indication of IVC filter placement in patients with a PE with a concomitant DVT include absolute contraindication to anticoagulation, failure of anticoagulation, a complication of anticoagulation, or DVT progression despite anticoagulation [8]. Our approach, despite not under any of these criteria, was deemed appropriate due to the high risk of further embolic events and a thorough literature review. A randomized controlled trial that portrays the benefits of IVC in conjunction with surgical and anticoagulation management in IPDE is lacking.

## Conclusions

A paradoxical embolism should be considered in patients who present with multiple signs of thromboembolic disease, including pulmonary and arterial circulation. Additionally, an IPDE is a rare finding and associated with a worse prognosis. Cardiac workup, including a TTE or a TEE, is a relevant consideration if presenting with stigmata of IPDE. However, this is hardly visualized in practice. Although the surgery is the mainstay of treatment for patients with an IPDE, the use of conjugate measures such as anticoagulation and PFO closure can mitigate risks associated with worsened outcomes, especially if there are current signs of embolization such as a PE or systemic embolization.
